# Psychiatrists And Clinical Psychologists*

**DOI:** 10.4103/0973-1229.27599

**Published:** 2006

**Authors:** Ajai Singh, Shakuntala Singh

Psychiatrists and clinical psychologists share an inevitable, if rather uneasy, relationship. So very much like a modern marriage. Can’t do without it, can’t get out of it. Both sides contemplate divorce often. Think of separation by mutual consent. Even keep threatening as they rave and rant. Have secret, and not so secret, flings on the side. But, like the proverbial homing bird, or the conservative Indian arranged marriage, have no option but to stick it out with each other.

Psychiatrists are otherwise good people. But that does not make them immune to handling clinical psychologists with the condescending tolerance and patronizing acceptance that teachers, for example, have towards rambling students. Or the rich have towards the poor. This does not take long to get converted into exasperation and smirky asides in the less charitable amongst the psychiatrists. Not that clinical psychologists are very helpful in motivating the psychiatrists to change for the better. For they, like most people in their position, over react and get aggressive when confronted with this attitude. And understandably so. However, it is time both realized their attitudes were not helpful either for mutual interaction, or growth of the Mental Health Movement at large.

We can understand why psychiatrists behave the way they do. They are exposed to this same condescending-patronising attitude from their own peers in the medical profession. Their medical colleagues have yet to develop a feeling of healthy respect for psychiatry. Psychiatrists, no doubt, feel this is unjustified, but their peers are still in a position to deny them the respect and acceptance they seek. What they get from their medical colleagues, they unwittingly pass on to their clinical psychologist colleagues. But understanding why it occurs does not absolve them of their responsibility to behave more rationally, rather than emotionally, with the latter.

The clinical psychologists, too, are not making it any easy for psychiatrists to change. They are often either protectively aggressive towards psychiatrists, or meekly submissive. Both rather immature responses. Their exasperation at neglect of the psychosocial while favouring the biological, which psychiatrists are at present so busy doing, manifests as aggressive criticism, and in clique formation, even joining the ranks of anti-psychiatry at times. While we can understand their anger, it is equally important for them to exercise caution so as not to overbalance and fall to the other extreme.

## The Dilemma

Psychiatry has always had, and will perennially have, to wrestle with the dilemma of being both a medical and a social science. It is condemned to the ambivalence that results from such a fate. We can rue it, and condemn and criticize it as much as we wish. But such is its fate, and such its destiny. It has no escape. So the biologically oriented try to explain every phenomenon important in psychiatry from a biological angle. Part of this approach means finding biological correlates of phenomena, which is a legitimate exercise. But part of it is also neglect, or disapproval, of the psychosocial approach itself. This often goes hand in hand with such an approach, almost unwittingly, and is the culprit. Whenever the biologically oriented psychiatrist tries to get the branch itself out of the psychological approach, he finds both his methods and his tools inadequate. The psychologically oriented amongst the psychiatrists are a ranting group at present, unhappy with the way the biologist is aggressively setting the psychiatry agenda, but equally powerless to change it, as of now. The psychiatrist, moreover, will always crave for acceptance from peers amongst his medical colleagues. And the more he is shunned by them, the more will he try to cosy up to them by proving how his branch is as biological, and therefore scientific, as theirs. If this is a tragedy according to you, well, it is one of gigantic proportions, and one to which no simple remedy comes to mind.

The eclectic approach, which we, and many others espouse, wherein the biological must blend with the psychosocial, and vice versa, has found favour with many so-called ‘balanced psychiatrists’. But that does not guarantee they decide the predominant research agenda of psychiatry today. That, at least at present, is firmly decided by the biologically oriented, with not a little help from industry sponsorship, which has an understandable stake in this whole paradigm shift towards the biological. Not that the psychiatrist researchers may necessarily be immune to this realization. But they may conveniently connive at such a movement. The reasons are not far to seek. The biological shift serves both parties very well indeed. The psychiatrist, to help establish his biological credentials, and therefore a respectable place amongst his medical colleagues and peers. And the pharmaceuticals, to firmly entrench themselves, for to support such biological shift takes care of their welfare and profits in no uncertain terms.

The clinical psychologist can watch these goings on with dismay and not a little impatience. He knows psychosocial factors are important. He sees their relevance in the patients (‘clients’) who do not recover, or recover only partially, with the psychiatrists’ drug regimen. He sees the psychiatrist unable to handle, or impatient in handling, the intrapersonal and interpersonal conflicts and frustrations of his patient. He sees the psychiatrist sucked into the vortex of tracing subtler nuances of drug biochemistry and drug-drug interactions. He sees bright minds wax eloquent over intricate neurophysiological and neurobiochemical mechanisms, many of them unproven flights of fantasy of equally bright minds hypothesizing on slender leads. Helped no end by the carrot of sponsorship of research as well as industry sponsored CMEs dangling in front always. So the researcher keeps busy talking of biological correlates. And the rest of the psychiatrists, equally impressed, do so to prove, to themselves more than any others, that their branch is properly biological, like the rest of medicine. The patient, meanwhile, who seeks care, is subjected to the wish fulfillment of such researchers. And he decides that the psychiatrist too, like the rest of the medical men, are busy looking into the intricacies and technicalities of his sickness process and perfecting their diagnosis, rather than mitigating his distress. While ostensibly solving patients’ problems, they are actually busy resolving their own.

## The Solution

There is a solution to this whole murky affair. However, it involves a real shift of perspectives, which can only come with some important realizations:

1. While biological correlates are important, as is linkage with the mainstream of medicine, and as is recognition from peers in the medical fraternity, the psychiatrist must know that he has no respite from the psychosocial, howsoever much he may try to blind himself to it. Hence, a better option for him would be to allow both the biological and psychosocial to flourish, and seek points of dynamic linkage between the two. Even as the biological grows, it respects and collaborates with the psychosocial. And vice versa. Psychiatry is in that unique a position as to prove the importance of the bio-psycho-social model relevant and appropriate to its branch. And, by extension, to prove its importance to the whole of medicine. A realization which is not current coin or on the research agenda of any branch of medicine at present, not that the right thinking in every branch, during their few moments of self-realisation, do not know it is indeed true. This challenge psychiatry is in danger of losing out on establishing, in its rather poor need to establish, and re-establish, credentials with its medical colleagues and research peers. Not that, *that* it not important. But it must be done on its terms, with its own unique method and approach. Like every other branch of medicine does: on its terms, with its own unique method and approach. We wonder whether there is any other branch of medicine as concerned with proving its biomedical credentials as psychiatry is. This is a sign of insecurity, and must change. This does not at all mean it delinks from the medical model. Far from it. It means it learns to be secure in its unique role of being both a medical and a social discipline.
2. The psychosocially oriented clinical psychologist, meanwhile, must not lose hope in his mental health colleague. He must present greater and more robust proof of the importance of psychosocial aspects in the genesis and progression of mental disorders. And be ready to stand the test of scientific scrutiny by rigorous experimental designs that the biologically oriented will insist on, both from psychiatry and even his colleagues in psychology. And stand his ground in scientific discussions based on evidence, getting neither unduly aggressive nor meekly submissive. The psychiatrist will then be helped resolve his dilemma, and come to terms with his unique position as a *medical man* in a *social science*. And actualize himself, and in the bargain, actualize the potentialities of his branch as well.

This is an important agenda before the clinical psychologist of today. And to make his task not so difficult that he gives up on him, is the task before the psychiatrist. Well, if you were to wonder why should the clinical psychologist show all this restraint and care for the psychiatrist when the latter is busy acting prodigal and arrogant, let it not be forgotten that this prodigal has immense potentialities, if brought on the right path by patience and understanding. And of course the occasional chastisement too. And what we envisage for the clinical psychologist we equally suggest for the psychosocially oriented psychiatrist. Rather these two must come together and get going to bring about a scientific renaissance of the psychosocial approach.

## Concluding Remarks

If we want the concept of mental health, and not just psychiatry, to succeed as a movement, the task is actually cut out for both parties who can make it succeed. In doing so, somewhere down the line, the greater task of actualizing the bio-psycho-social approach to medicine, and the related concept of holistic patient welfare, will become a goal that appears within sight. And medicine, not just to treat distress and disability, but also to promote well-being will not remain only a mirage it is at present.

Will both sides realize this, and rise to the challenge?

